# Searching for the Mechanical Fingerprint of Pre-diabetes in T1DM: A Case Report Study

**DOI:** 10.3389/fbioe.2020.569978

**Published:** 2020-09-29

**Authors:** Flavio Di Giacinto, Linda Tartaglione, Matteo Nardini, Alberto Mazzini, Sabrina Romanò, Gaetano Emanuele Rizzo, Massimiliano Papi, Marco De Spirito, Dario Pitocco, Gabriele Ciasca

**Affiliations:** ^1^Dipartimento di Neuroscienze, Sezione di Fisica, Università Cattolica del Sacro Cuore, Rome, Italy; ^2^Fondazione Policlinico A. Gemelli IRCCS, Rome, Italy; ^3^Diabetes Care Unit, Catholic University School of Medicine and Fondazione Policlinico Universitario “A. Gemelli” Istituto di Ricovero e Cura a Carattere Scientifico (IRCCS), Rome, Italy

**Keywords:** diabetes, pre-type 1 diabetes, AFM, cell mechanics, biomechanics, red blood cells, standard linear solid model, viscoelasticity

## Abstract

We report the case of a 38 year-old Caucasian man enrolled in a study aimed at investigating the physical properties of red blood cells (RBCs) using advanced microscopy techniques, including Atomic Force Microscopy (AFM). At the time of his first enrolment in the study, he had normal Fasting Plasma Glucose (FPG) values, a BMI of 24.1, and no other symptoms of diabetes, including fatigue, high triglycerides, low HDL cholesterol, and altered inflammatory and corpuscular RBC indices. The subject reported no family history of diabetes, obesity, and cardiovascular diseases. Despite his apparently healthy conditions, the biomechanics of his RBCs was altered, showing increased values of stiffness and viscosity. More than 1 year after the mechanical measurements, the subject was admitted to the Operational Unit of Diabetology of the Policlinico Gemelli Hospital with high blood glucose and glycosylated hemoglobin (HbA1c) levels and diagnosed with type 1 diabetes (T1DM). Here, we show these data, and we discuss the hypothesis that RBC mechanical properties could be sensitive to changes occurring during the pre-diabetic phase of T1DM.

## Introduction

Red Blood Cells (RBCs) possess extreme mechanical properties that allow them to undergo large deformations when they pass through the microcirculation. On the nanoscale, the mechanical properties and shape of RBCs arise from a characteristic spectrin-network representing the cell skeleton, which is tethered to the lipid membrane bilayer by integral and peripheral proteins (Diez-Silva et al., 2010). This complex network, which also changes dynamically according to the shear stress, contributes to maintaining cell integrity after repeated deformations. Such extreme deformability is significantly altered in many clinical conditions, suggesting that studying RBC biomechanics can be a source of potential biomarkers for many pathologies (Perrotta et al., 2008; Fedosov et al., 2011; Tomaiuolo, 2014; Ciasca et al., 2015). In this context, Atomic Force Microscopy (AFM) has been proven to be an effective tool to study erythrocytes biomechanics at the nanoscale level. In a previous study, we used AFM to investigate the effects of type 2 diabetes mellitus (T2DM) on RBC mechanical properties, demonstrating an increase in cell stiffness and hysteresis for the T2DM cohort compared to the healthy subjects (Ciasca et al., 2015). Such a stiffening was consistent with the results previously discussed in the papers of Pretorius and co-workers (Buys et al., 2013; Pretorius and Kell, 2014; Pretorius et al., 2015; Visser et al., 2017) and with similar AFM studies summarized in Table 1 (Gillies and Prestidge, 2004; Lekka et al., 2005; Dulińska et al., 2006; Fornal et al., 2006; MacIaszek et al., 2011; Girasole et al., 2012; Li et al., 2012; Lamzin and Khayrullin, 2014; Ciasca et al., 2015; Pretorius et al., 2015; Zhang et al., 2015; Barns et al., 2017). In the recent paper from Loyola-Leyva et al. (2018), mechanical measurements carried out on RBCs obtained from diabetic and control subjects with techniques other than AFM are also reviewed.

**Table 1:** Red Blood Cell rigidity in healthy and diabetic subjects as measured in previous Atomic Force Microscopy experiments.

**References**	**Subjects**	**Average E (kPa)**	**Rate**	**Preparation**
Lekka et al. (2005)	*N* = 13 Healthy subjects ramya *N* = 19 Diabetes Mellitus	4.9 ± 0.5 ramya 11.3 ± 1.5 (Estimated from the paper)	Not indicated	Dried cells (not fixed)
Girasole et al. (2012)	Healthy subjects (number not reported)	Ranges between 75–115 KPa	Not indicated	Fixed cells
Gillies and Prestidge (2004)	Healthy subjects (number not reported)	Ranges between 1, 27–7, 22	Ranges between 0.6–2.8 μm/s	Cells resuspended in PBS solution. Not Fixed
Dulińska et al. (2006)	Healthy subjects (number not reported)	26	Not indicated	Fixed cells (0.5% glutaraldehyde) resuspended in PBS
Li et al. (2012)	Healthy subjects (number not reported)	Ranges between 0.1–0.2	Not indicated	Cells resuspended in Hank's balanced salt solution (HBSS). Not Fixed
Maciaszek and Lykotrafitis (2011)	Healthy subjects (Number not reported)	Monomodal distribution peaked at 1.1 ± 0.4	Not indicated	Cells resuspended in PBS solution. Not fixed
Lamzin and Khayrullin (2014)	*N* = 40 Healthy subjects	1.81 ± 0.4	Not indicated	Dried cells. Not Fixed
Zhang et al. (2015)	*N* = 10 Healthy subjects ramya *N* = 10 Diabetes mellitus	0.31 ± 0.03 ramya 24.83 ± 0.61	Not indicated	Cells resuspended in Hank's balanced salt solution (HBSS). Not Fixed
Barns et al. (2017)	*N* = 4 Healthy subjects	7.57 ± 3.25	1 μm/s	Fixed cells (1.0% glutaraldehyde) resuspended in PBS
Fornal et al. (2006)	*N* = 8 Healthy subjects ramya *N* = 7 Diabetes mellitus	4.4 ramya 14.4	Not Available	Not Available
Ciasca et al. (2015)	*N* = 10 Healthy subjects ramya *N* = 5 Diabetes mellitus	1.82 ± 0.20 ramya 2.52 ± 0.58	5 μm/s ramya 5 μm/s	Cells resuspended in PBS solutions. Not Fixed
Measured in Pretorius et al. (2015) and reviewed in Loyola-Leyva et al. (2018)	*N* = 25 Healthy subjects ramya *N* = 69 Diabetes mellitus	46710 ± 39210 ramya 56483 ± 6418	QNM frequency between 430 and 516 kHz	Fixed cells (formaldehyde followed by a dehydration)

Despite a consensus exists that the hyperglycemic and inflammatory conditions in full-blown diabetes induce severe alterations in the RBC biomechanical response, it is still unclear at what stage of the diabetes progression the rheological and mechanical characteristics begin to change as well as to what extent they change in the early stages of the disease.

In this regard, it is relevant to stress that previous AFM studies on RBC biomechanics in diabetes were mainly based on the determination of Young's modulus *E*, thus implicitly assuming a purely elastic behavior. Despite this is often a crude approximation, it is still possible to observe detectable changes between pathological and physiological conditions when mechanical alterations are relevant. However, the interplay of elastic and viscous contributions is known to affect the determination of *E* in a complex and often unpredictable fashion, thus masking the presence of more subtle modifications, which are more likely to occur in the early stages. In the latter case, decoupling viscous and elastic terms in the framework of the standard linear model (SLS) has often revealed an effective approach. In this context, advanced AFM application can be found in references (Moreno-Flores et al., 2010; Rianna and Radmacher, 2016, 2017; Heydarian et al., 2020; Zhang et al., 2020). It is also worth noting that the optimum loading conditions to retrieve Young's modulus *E* under linear elastic assumption has been systematically investigated in the recent paper of Kontomaris et al. (2019).

In this study, we describe a case report of a subject enrolled in a previous investigation on the physical properties of RBCs. The subject initially included in the control group, showed normal Fasting Plasma Glucose (FPG) levels and had no evident symptoms of diabetes, but the analysis of the biomechanical properties of his RBCs showed altered values of stiffness and viscosity if analyzed in the framework of the SLS model. More than 1 year after the AFM measurements, the subject started to show symptoms of diabetes and he was diagnosed with Type 1 Diabetes Mellitus (T1DM). In this work, we show these data, and we discuss the hypothesis that RBC mechanical properties can be sensitive to subtle changes in the glucose concentration in blood, even in the early stage of diabetes.

## Case Description

The measures performed on a total of 20 healthy donors aged between 26 and 59 years (mean age: 42 years, standard deviation: 7 years) carried out in previous experiments were screened and considered for the inclusion in the present analysis. Blood samples were obtained by venipunctures at the Policlinico Gemelli of Rome according to the institutional bioethics code (prot. 1457). Among the inclusion criteria, there was a normal blood glucose level and a Body Mass Index (BMI) between 18.5 and 24.9. It is worth stressing that the subjects were not recruited specifically to study the mechanical properties of red blood cells in pre-diabetes. In March 2019, one of the subjects enrolled in the study, a 38 year-old man with a BMI of 22.4, was admitted to the Operational Unit of Diabetology of the Policlinico Gemelli Hospital with high blood glucose and glycosylated hemoglobin (HbA1c) levels. The subject presented with significant visual impairment. No previous diabetes or heart disease history was reported and at the time of his first enrolment in the study, the subject showed a normal blood glucose level (94 mg/dl) and a BMI of 24.1. After admission to our department, the relevant examinations were completed, and the subject was diagnosed with T1DM. At discharge, the prescribed blood glucose regulation regimen included subcutaneous injections of insulin glargine before sleep and of insulin aspart before breakfast, lunch, and supper. The subject immediately adhered to the prescribed therapy, as demonstrated by a monotonous decrease in his HbA1c levels in the subsequent months, until reaching normal values.

His blood examination at the time of the enrolment in the study (November 2017) and at the time of his T1DM diagnosis (March 2019) are compared in Table 2.

**Table 2:** Subject's Blood Tests at the first enrolment in the study and after receiving his T1DM diagnosis.

**Blood test**		**First enrolment(November 2017)**	**After diagnosis(March 2019)**	**Range**
Hemoglobin	g/dL	15.8	15.3	13–17
Hematocrit	%	46	46.4	40–50
Red blood cell	x10^12^/L	5.35	5.24	4.5–55
Mean cell volume (MCV)	fL	86.1	88.5	83–101
Mean cell hemoglobin (MCH)	Pg	29.5	29.3	27–32
Mean cell Hb concentration (MCHC)	g/dL	34.3	33.1	31.5–34.5
Red blood cell distribution width (RDW)	%	13.2	13.6	11.5–14.5
Platelets	x10^9^/L	280	317	150–450
MPV	fL	7.6	7.8	6.8–10
WBC count	x10^9^/L	6.32	4.74	4.0–10
Cholesterol, total	mg/dL	167	151	130–200
HDL cholesterol	mg/dL	44	46	>40
LDL cholesterol	mg/dL	110	93	<130
Tryglicerides	mg/dL	108	56	20–170
Glucose	mg/dL	94	161	65–110
Glycosylated hemoglobin (HbA1c)	mmol/mol	N.A.	91	23–41

## Materials and Methods

Blood samples were processed as described elsewhere (Ciasca et al., 2015). Measures were performed with a JPK NanoWizard II AFM, equipped with Si-cantilevers with conical tip (nominal spring constant 0.03 N/m), at room temperature and in physiological solution (Ciasca et al., 2015; Perini et al., 2019). Mechanical properties were studied through the acquisition of Force-Distance (FD) cycles and Force Relaxation (FR) curves.

Before discussing the paper experimental design, a *caveat* is necessary. As stated above, the experiment was not specifically conceived to study the RBCs mechanical modifications occurring in the pre-diabetic patient, as we weren't expecting that the subject–initially included in a control group for a different experiment–more than 1 year later would have received a T1DM diagnosis. This event, on the one hand, put us in the rare condition of being able to observe that such alterations might occur far before diagnosis, on the other hand, it introduced inherent statistical limitations, also specifically related to the AFM technique. As shown in Table 1, the RBCs mechanical properties measured with AFM undergo very large variation in literature, partly because of the sample biological variability, but also depending on the cell preparation and the AFM experimental configuration (measurement mode, tip shape, indentation speed, and indentation force). Therefore, to allow for the direct comparison of AFM data between the subject and the controls, we had to select a subset of consistent measurements within a much larger database obtained in different experiments carried out at different times.

**Figure 1:**
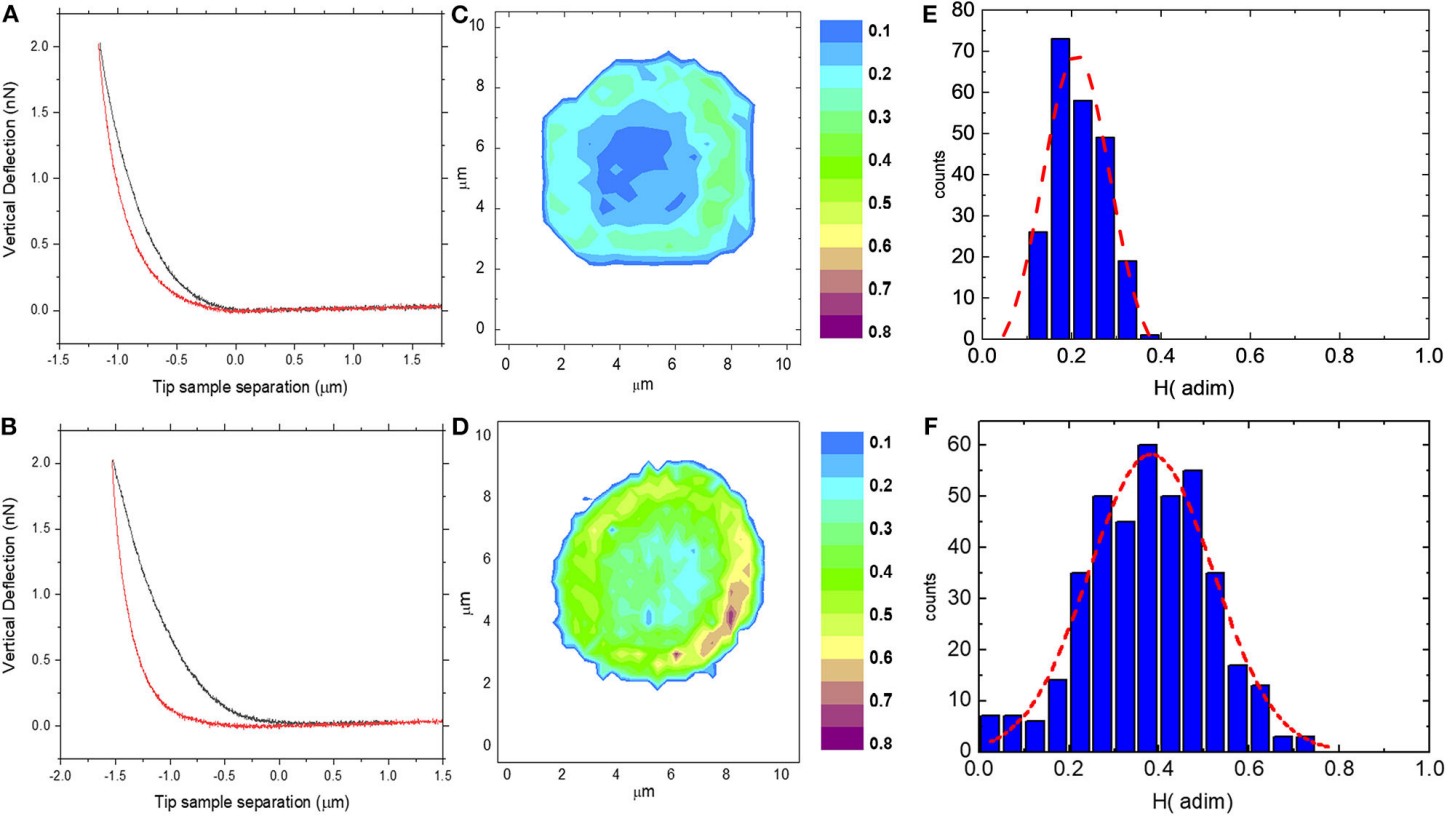
Two representative Force-Distance (FD) cycles acquired on a control subject **(A)** and on the pathological subject **(B)**; two representative erythrocyte H maps acquired on control **(C)** and on the pathological subject **(D)** with the corresponding histogram of H values **(E,F)**.

FD cycles were analyzed to obtain the AFM hysteresis *H*, which is the perceptual energy dissipated during indentation (Figures 1A–F). *H* is computed as the difference between the integral of the approach curve F_A_(δ) and the retract curve F_R_(δ), normalized as follows (Ciasca et al., 2016, 2019; Minelli et al., 2018; De-Giorgio et al., 2019; Mazzini et al., 2020):

(1) $$\begin{array}{r} H=\frac{\int_{0}^{\delta_{\max}}F_{A}\left(\delta \right)d\delta -\int_{0}^{\delta_{\max}}F_{R}\left(\delta \right)d\delta }{\int_{0}^{\delta_{\max}}F_{A}\left(\delta \right)d\delta }=\frac{A_{E}-A_{R}}{A_{E}} \end{array}$$

We set an indentation force of 2 nN and a speed during indentation of 5 μm/s. Two sampling strategies were adopted: elasticity maps were measured on 10 single cells with a resolution ranging from 32 × 32 to 8 × 8 pixels, allowing us to obtain functional images of the cell mechanical properties; larger Petri regions, typically (100 × 100) μm^2^ in size, were investigated to increase statistical sampling. The subject measurements were compared with values obtained in previous experiments, averaging the results obtained on 15 controls subjects measured with an indentation force of 2 nN and an indentation rate ranging from 4 to 6 μm/s.

**Figure 2:**
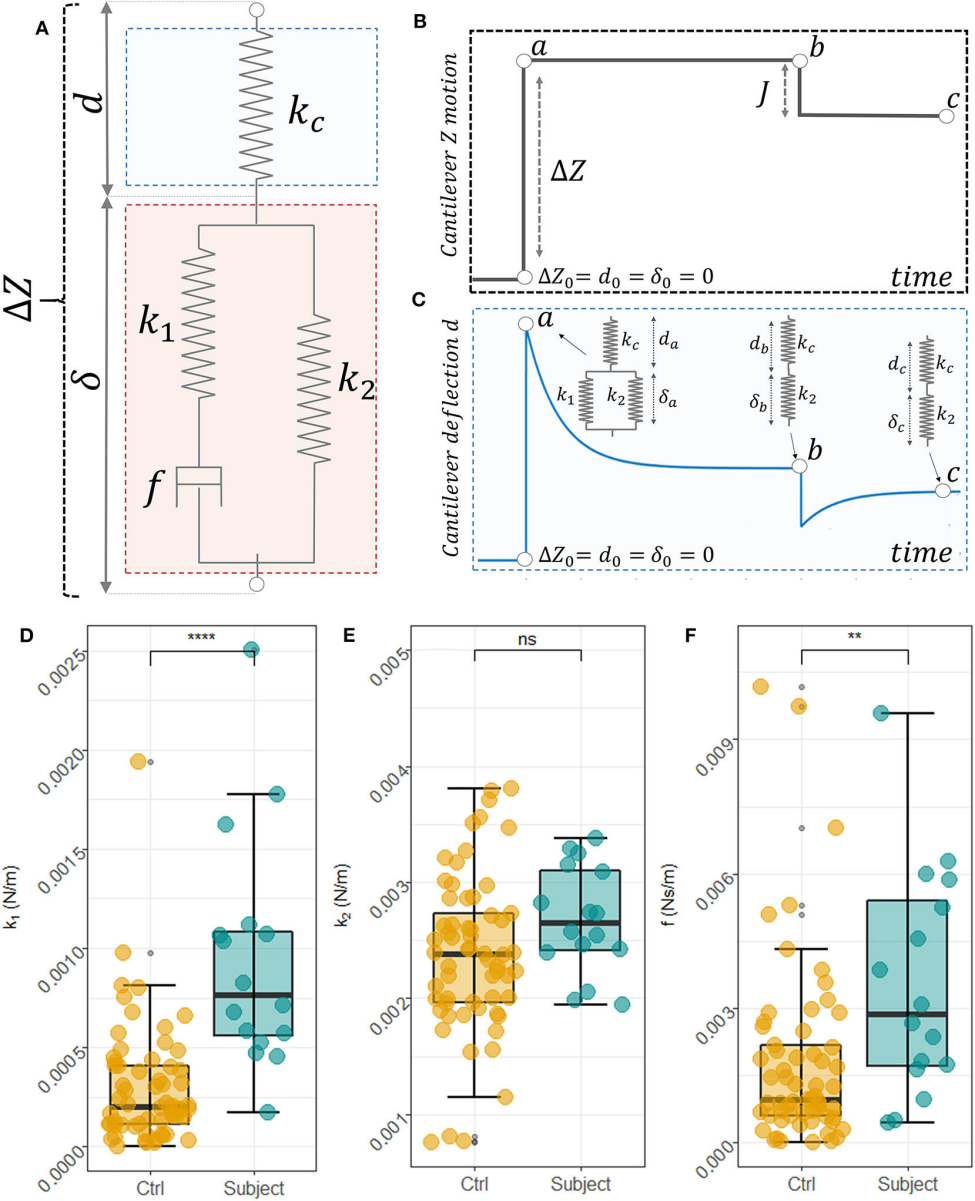
Scheme of the SLS model used to describe RBCs mechanical behavior and interaction with AFM **(A)**. Schematic view of the cantilever position overtime during the measurements **(B)** and the corresponding cantilever deflection **(C)**. In the specific equilibrium points point *a, b*, and *c* the mechanical behavior of the sample and the cantilever is represented in the insets of **(C)**. Box plot analysis of the obtained SLS parameters, namely *k*_1_ **(D)**, *k*_2_ **(E)**, and the *f* **(F)** for control subjects and the pathological one. An unpaired two-samples Mann Whitney *U*-Test was used to compare data.

Following Rianna and Radmacher (2017), we analyzed FR curves in the framework of the Standard Linear Solid (SLS) model (Figure 2A), which is a way of representing samples as a linear combination of two elastic springs (*k*_1_ and *k*_2_) and a viscous dashpot (*f*). To this purpose, we define the following variables: *Z* referred to as the cantilever position, *d* the cantilever deflection, and δ the sample indentation. The variable δ, which contains the mechanical information about the sample, cannot be measured directly, but it has to be inferred from the position *Z* of the cantilever over time and the corresponding cantilever deflection, *d*. For these variables, the following relationship can be established: *Z* = *d*+δ → δ = *Z*−*d* (Figure 2A). In our configuration, the typical time-behavior of *Z* and *d* are schematically represented in Figures 2C,D, respectively. At the contact point, immediately before indenting the sample, we can set *Z*_0_ = *d*_0_ = δ_0_ = 0, as the cantilever is not deflected, the sample is not indented and the *Z* = 0 position can be chosen arbitrarily. During the approach and indentation phase, we exploited a high indentation speed (35 μm/s) to avoid possible sample relaxation during indentation, before the target force is reached (point *a*, in Figures 2B,C). In this fast indentation phase, we can assume that the viscous dashpot has had no time to respond and, thus, the sample in the point *a* behaves just like the parallel between *k*_1_ and *k*_2_, as schematically represented in the inset of Figure 2C. Then, a dwell phase at constant height is imposed, where the piezoelectric extension remains constant (Figure 2B) and the sample is allowed to relax. In this phase, $Z=cost\to \overset{∙}{\delta }=-ḋ$ and the following differential equation establishes a relation between cantilever deflection and sample indentation:

(2) $$\begin{array}{r} k_{c}d+\frac{f}{k_{1}}\left(k_{c}ḋ\right)=k_{2}\delta +f\frac{\left(k_{1}+k_{2}\right)}{k_{1}}\overset{∙}{\delta } \end{array}$$

where *k*_*c*_ is the cantilever elastic constant that was measured before each experiment with the thermal calibration method implemented in the instrument software. In the constant height mode $\left(Z=cost\to \dot{\delta }=-\dot{d}\right)$, this equation can be written as $d\left(t\right)=\frac{k_{2}}{\left(k_{2}+k_{c}\right)}Z-\tau_{c}ḋ$, and solved as follows:

(3) $$\begin{array}{r} d\left(t\right)=Ae^{-\;\frac{t}{\tau_{c}}}+d_{b} \end{array}$$

with $\tau_{c}=\frac{f\left(k_{1}+k_{2}+k_{c}\right)}{k_{1}\left(k_{c}+k_{2}\right)}\;$, $A=\left(d_{a}-\frac{k_{2}}{\left(k_{c}+k_{2}\right)}\cdot \Delta Z\right)$ and $d_{b}=\frac{k_{2}}{\left(k_{c}+k_{2}\right)}\cdot \Delta Z$, where *d*_*a*_ and *d*_*b*_ are the cantilever deflections at points *a* and *b*, respectively (Figure 2C). *A*, τ_*c*_, and *d*_*b*_, can be obtained fitting the measured FR curves with Equation (3) and used to retrieve *k*_1_, *k*_2_, and *f*. Unfortunately, these quantities depend on *Z* and *d*_*a*_, which are likely to be affected by a large experimental error in our set-up. To overcome this problem, a height step is applied to the cantilever when the first relaxation is completed, retracting the tip of a quantity *J* that is exactly known (Figure 2B). Being a pure elastic body, the cantilever instantly follows this variation, while the cell initially maintains its deformed state and then relaxes until a new equilibrium in point *c* is reached (Figure 2B). In this point, the following equilibrium equation can be written: _*k*_*c*_*dc*_ = *k*_2_(*Z*−*J*−*d*_*c*_), where *d*_*c*_ is the measured deflection at the new equilibrium position. As shown in the inset of Figure 1C, a similar equation can be written for the point a: *k*_*c*_*d*_*a*_ = (*k*_1_+*k*_2_)(*Z*−*d*_*a*_). Combining the latter five equations, we can get rid of *Z* and *d*_*a*_ and we can find a mathematical expression for the SLS parameters (Equations 4):

(4) $$\{\begin{array}{c} k_{1}=\frac{A\left(k_{c}+k_{2}\right)k_{2}}{\left(k_{c}d_{b}-k_{2}A\right)}\\ k_{2}=\frac{k_{c}(d_{b}-d_{c})}{\left(J+d_{c}-d_{b}\right)}\\ f=\tau_{c}\frac{k_{1}\left(k_{c}+k_{2}\right)}{\left(k_{1}+k_{2}+k_{c}\right)} \end{array}$$

The SLS parameters were obtained for each measured cell, separately. A statistical comparison between the cells measured on the subject and the controls was performed with the independent 2-groups Mann-Whitney *U*-test implemented in the *stat_compare_means()* function of the R package *ggpubr*.

## Results

In Figures 1A,B, two representative FD cycles acquired on a control subject and the pathological subject are reported, respectively. A larger area between the approach and the retract curve, which corresponds to a higher AFM hysteresis (*H*) value, can be observed for the pathological subject compared to the control one. Due to the heterogeneity of biological samples, a single point measurement is not representative of the whole-cell mechanical response. Therefore, we probed the local response of RBCs through the acquisition of FD cycles at different positions over the cell surfaces and we computed the *H* value in each position. In Figures 1C,D, we reported two representative erythrocyte *H* maps acquired on a control (*c*) and the pathological subject (*d*). *H*-values are approximately in the range 0–0.4 for the control subject (*d*) and 0–0.75 for the pathological one, suggesting that larger energy is dissipated in the latter case. The map of the control subject shows a cylindrical symmetry, with higher *H* values at the cell periphery than in its center. The observed cylindrical distribution seems to reflect the typical biconcave shape of healthy erythrocytes. In the pathological case, the nanoscale map appears to be brighter than that of the healthy RBCs, confirming that more energy is dissipated during indentation. Moreover, the *H* difference between the cell center and its edge is less pronounced than in the previous case. The two maps are quantitatively compared with the corresponding *H* histogram (Figures 2E,F, respectively). A Gaussian curve was fitted to data. The frequency distribution of the control cell is peaked at ~0.17 with a mean *H* of 0.21 ± 0.01. Data are expressed as mean ± SEM. A shift of the frequency distribution at larger *H* values can be observed in the pathological case, with a peak at ~0.4 and an average *H* of 0.38 ± 0.02. In Supplementary Figure 1, we report a box-plot analysis of all the cells measured on the subject. Average cell values are indicated with a red diamond. These measurements are compared with the mean ± SEM interval for controls (see Materials and Methods). The observed changes in *H* values hint to an increased cell viscosity in the pathological case or an increased cell fragility. Unfortunately, *H* does not provide a quantitative measure of sample viscosity, because of the interplay between elastic and viscous contributions that affect the FD-cycle shape in a complex and hardly predictable fashion.

To overcome these limitations, we designed a specific sequence of FR curves that allowed us to deconvolve elastic and viscous contributions (Figure 2). Based on Rianna and Radmacher (2016, 2017), we analyzed these curves in the framework of the SLS model, thus describing the sample as a linear combination of two elastic springs (*k*_1_ and *k*_2_) and a viscous dashpot (*f*). The model is schematically represented in Figures 2B,C and discussed in Materials and Methods according to Equations (2–4). These equations permit to obtain the sample indentation δ over time, which contains the mechanical information on the sample, from the knowledge of the cantilever position *Z* and deflection *d* over time, schematically represented in Figures 2B,C, respectively. In Supplementary Figure 2, we show two average FR curves for the controls and the subject. Curves are reported as mean ± 95% CI. From the analysis of Supplementary Figure 2, a difference between the pathological subject and the control ones can be observed, with the former showing a deeper and slower time relaxation than the latter. To quantify this difference, we analyzed data using Equations (2–4), which allowed us to retrieve the SLS parameters, namely the elastic constants *k*_1_ and *k*_2_, and the damping element *f*. A box plot analysis of these parameters is reported in Figures 2D–F, respectively. Each dot corresponds to the measurement performed on a single cell. Raised values of all three parameters can be observed for the subject's cells compared to the controls' ones. The result of a non-parametric independent 2-groups Mann-Whitney *U*-test is superimposed to each plot, showing the presence of a significant difference in the parameters *k*_1_ and *f*. These differences suggest that cells obtained from the subject are stiffer and more viscous than that measured on controls.

Being the present work a single case report study, and being the AFM an inherently low throughput technique, these results have to be considered preliminary. Therefore, in the Discussion section, we attempt to provide a biochemical and proteomic rationale behind these changes to connect the physics of erythrocyte's membrane with the biologic alterations of proteins involved. Due to the preliminary nature of these results, we also attempted to evaluate possible causes other than the pre-diabetic phase that might have induced the observed changes.

## Discussion

Type-1 diabetes mellitus is a complex disorder characterized by the autoimmune destruction of β-cells, which causes a loss of insulin production, thus impairing glucose transport into cells and leading to consequent hyperglycemia. According to a widely accepted hypothesis, T1DM onset occurs when more than 80% of the β-cells have been destroyed. Although the peak incidence of T1DM is during adolescence, experimental evidence suggests that 5–10% of all adults diagnosed with T2DM might have T1DM (LADA: latent autoimmune diabetes in adults) (Simell et al., 2010). Despite Pre-diabetes is generally linked with T2DM, it is likely that also T1DM individuals had a pre-diabetic phase with signs and symptoms of diabetes before the diagnosis. A better understanding of these signs and symptoms is a fundamental and priority step, as the pre-diabetic period provides a window of opportunity for early intervention (Das, 2015).

The pre-diabetic phase is usually characterized by a low tolerance to glucose and it is often asymptomatic. In T2DM patients this is often associated with obesity, dyslipidemia with high triglycerides and/or low HDL cholesterol and hypertension (American Diabetes Association, 2017). The subject's lipid panel (Table 2) does not suggest the presence of dyslipidemia, neither shows altered triglycerides and cholesterol levels or other factors hinting at the presence of the metabolic syndrome. His BMI was lying in the normal range. Interestingly, he presented with hypertension under treatment, further supporting the idea that hypertension is associated with a greater risk of diabetes, beyond that explained by other risk factors (Wei et al., 2011).

It is widely accepted that reactive oxygen species are increased by hyperglycemia (King and Loeken, 2004), and RBCs are one of the cell types that are particularly vulnerable to oxidative stress (Buys et al., 2013; Pretorius, 2013; Pretorius and Kell, 2014; Visser et al., 2017). Alterations in the overall cell shape, size, membrane roughness and lipid composition, elastic and rheological properties, have been deeply documented in T2DM patients (Buys et al., 2013; Hoffmann et al., 2015; Maulucci et al., 2017; Bianchetti et al., 2019). In T2DM however, RBCs remain in a hyperglycemic environment throughout their entire lifespan, therefore the changes in their structure and flow properties are particularly evident, resulting also in macroscopic alterations of corpuscular and inflammatory indices, as provided by conventional blood tests (Chehaibi et al., 2016; Alamri et al., 2019). Conversely, RBCs in the pre-diabetic phase are likely exposed to a moderate and oscillating level of hyperglycemia, which might induce less evident and more subtle modifications at the level of the cell structure. Consistently, the patient showed no alteration of corpuscular and inflammatory indices and a fasting glucose level in the normal range. This result is highly interesting and deserves a more in-depth study as it suggests that studying the biomechanical response of red blood cells might be more effective than looking at FPG and conventional RBC indices for the detection of early signs of the pre-diabetic phase. In this regard, one has to consider that oxidative stress is known to alter RBC biomechanics through different pathways and at different cell levels: the cytoskeletal proteins of RBCs from diabetic patients are heavily glycosylated and spectrin is oxidatively damaged; several lipids, including free cholesterol, sphingomyelin, and phosphatidylcholine on the outer surface of the phospholipid bilayer are significantly decreased; surface proteins are heavily glycosylated, suggesting an altered connection between the cytoskeletal matrix and band 3 and 4 integral proteins (Buys et al., 2013; Pretorius, 2013; Pretorius and Kell, 2014; Visser et al., 2017). Other possible alterations in the interaction between integral membrane proteins and cytoskeletal components have been recently reviewed in the paper of Bosman (2016) and Jiang et al. (2003) have documented a total of 42 RBC proteins differentially expressed in the RBC membranes of T2DM patients compared to controls, including flotillin-1 that is believed to contribute to stimulating the activation of glucose transporter 4 in response to insulin, and syntaxin 1C and arginase that might be liked to changes in red blood cell volume and morphology. Such a systemic response may provide a rationale behind the hypothesized sensitivity of biomechanical properties to the early changes in the pre-diabetic phase. In this regard, a caveat is necessary, because the subject's HbA1c was not measured at the time of his first enrolment in the study. Therefore, we cannot exclude a causative link between this parameter and the measured mechanical alterations and we cannot exclude as well that the subject wasn't indeed in the pre-diabetic phase, but rather he had a misdiagnosed full-blown diabetes.

The RBC stiffening measured in Figure 2E can be discussed also considering the AFM results reported in Table 1, which summarizes several studies carried out on RBC obtained from healthy and diabetic subjects or healthy subjects alone. This table highlights that the absolute values of mechanical parameters strongly depend on the method used during the measurements, on the sample pre-processing, and the specific technique used. Nevertheless, all the studies, independently on the specific method or technique, revealed a general stiffening of RBC in diabetes, which is consistent with our results.

Moreover, it is well-known that cells can adapt to their mechanical environment. For example, metastatic cancer cells may alter their cortex stiffness to resist the shear flow, when intravasate into blood vessels and capillaries, giving rise to metastasis (Kumar and Weaver, 2009; Ciasca et al., 2016). Despite the relationship between erythrocyte biomechanics and hypertension has never been reported in the literature, at present, we have not enough information to exclude that the measured biomechanical alterations are an adaptative response to high blood pressure and not an early sign of diabetes.

## Conclusion

In this study, we unveiled alterations in the mechanical properties of erythrocytes obtained from a type 1 pre-diabetic patient, ~1 year before his diagnosis. Interestingly, at the time of his first enrolment in the study, the patient showed no evident symptoms of diabetes, including normal FPG levels. We exploited a specific AFM experimental setup based on the application of the SLS model to quantify changes in RBC deformability, which allowed us to detect a higher RBC viscosity and rigidity. The results here presented suggest that RBC biomechanics could be particularly sensitive to pathological changes occurring in the early stages of diabetes. The difficulty of enrolling other similar cases–T1DM pre-diabetic patients with no evident symptoms–did not allow us to enlarge the statistical sampling and strengthen our results. However, the case here reported can represent a starting point and a shred of evidence for further studies in this direction.

## Data Availability Statement

The raw data supporting the conclusions of this article will be made available by the authors, without undue reservation.

## Ethics Statement

The studies involving human participants were reviewed and approved by Ethics Committee of Policlinico Universitario Agostino Gemelli IRCCS at comitato.etico@policlinicogemelli.it or Tel. 06-30155556 06-30156124. The patients/participants provided their written informed consent to participate in this study.

## Author Contributions

GC and DP conceived the work. GC, DP, MP, and FD wrote the paper. MN, AM, and SR acquired measurements and analyzed data. MD and DP supervised the work. FD and GC developed the mechanical model. GC programmed the homemade software for the analysis of the AFM curves. LT, GR, and DP recruited subjects, collect samples, and provided the biochemical analysis. All authors contributed to the article and approved the submitted version.

## Conflict of Interest

The authors declare that the research was conducted in the absence of any commercial or financial relationships that could be construed as a potential conflict of interest.

## Supplementary Material

Supplementary Figure 1 Box plot analysis of the measures performed on the 10 cells measured on the subject. Horizontal lines represent the Mean ± SEM measured on healthy controls.

Supplementary Figure 2 Average time-dependent force relaxation sequences calculated using FR curves acquired on the RBC center on healthy subjects **(A)** and on the pathological one **(B)**. The corresponding 95% confidence bands about the mean are also reported.
