# Supplementary figures and images for: Searching for the Mechanical Fingerprint of Pre-diabetes in T1DM: A Case Report Study
Source: Front Bioeng Biotechnol. 2020 Sep 29;8:569978. doi: 10.3389/fbioe.2020.569978 (PMC7552738; doi:10.3389/fbioe.2020.569978)

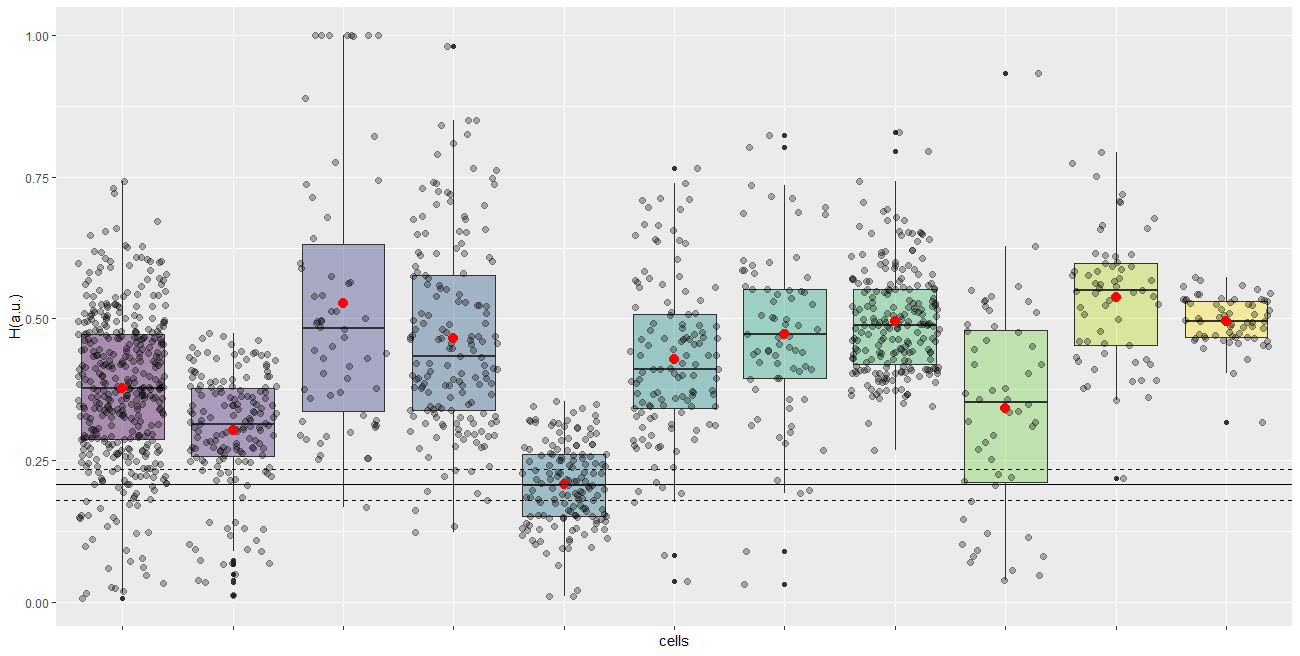

Supplement: Supplementary Figure 1 — Box plot analysis of the measures performed on the 10 cells measured on the subject. Horizontal lines represent the Mean ± SEM measured on healthy controls. [file Image_1.PNG]

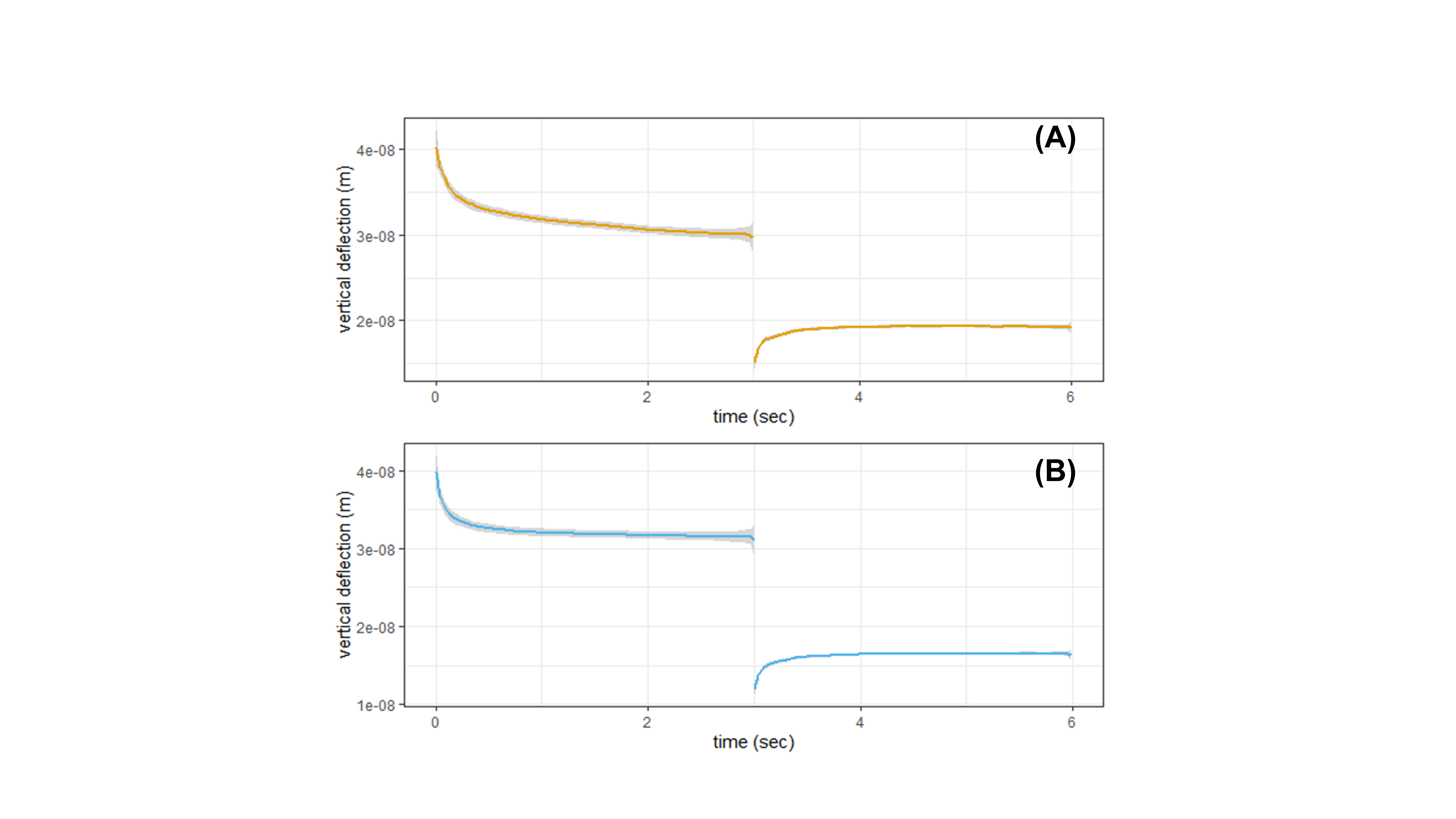

Supplement: Supplementary Figure 2 — Average time-dependent force relaxation sequences calculated using FR curves acquired on the RBC center on healthy subjects (A) and on the pathological one (B). The corresponding 95% confidence bands about the mean are also reported. [file Image_2.PNG]
